# *ets-10p::gfp* expression is predictive of dauer formation in *daf-16; daf-7* larvae

**DOI:** 10.17912/micropub.biology.001358

**Published:** 2024-10-22

**Authors:** Juanez T Lindsay, Matthew J Wirick, Xantha Karp

**Affiliations:** 1 Department of Biology, Central Michigan University; 2 Biochemistry, Cell and Molecular Biology Program, Central Michigan University; 3 Department of Biology, Central Michigan University, Mount Pleasant, MI 48859

## Abstract

In adverse conditions, *Caenorhabditis elegans* larvae can enter the alternative L2d stage. If conditions remain poor, L2d larvae can molt into stress-resistant dauer larvae. The FOXO ortholog *daf-16* promotes dauer formation, but *daf-16* mutants can enter dauer with incomplete penetrance in combination with a mutation in *daf-7* /TGFβ. The degree to which *daf-16 ; daf-7* larvae enter L2d is unknown. Here we show that many *daf-16 ; daf-7* mutants express intermediate levels of the *ets-10p::gfp* L2d marker, suggesting incomplete entry into L2d. Furthermore, lack of *ets-10p::gfp* expression early in the second larval stage partially predicts which *daf-16 ; daf-7* larvae will bypass dauer formation.

**Figure 1. daf-16; daf-7 larvae express intermediate levels of the ets-10p::gfp L2d marker prior to dauer formation:**
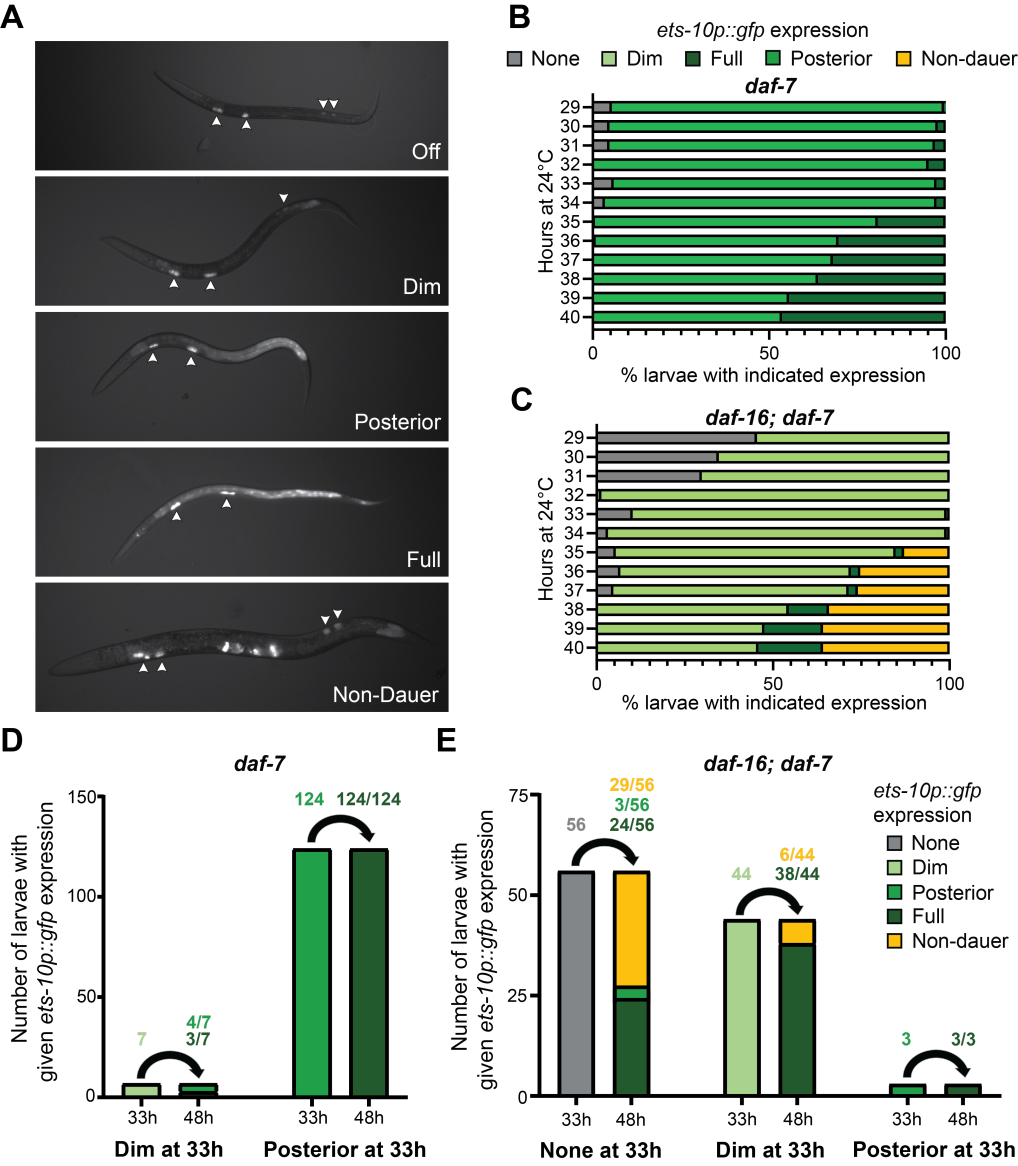
(A) Representative images for categories assigned in panels B-E. “Off” indicates that expression is undetectable; this lack of expression was characteristic of continuously developing L2-staged larvae (Shih et al. 2019). “Dim” indicates expression is faintly visible in the posterior intestine. “Posterior” indicates robust expression in the posterior intestine but no expression in the anterior. “Full” indicates strong expression throughout the intestine. “Non-dauer” indicates expression in the spermatheca and vulva, but at most weak expression in the intestine. Note that expression from the injection marker, *ofm-1p::rfp,* bleeds through the broad GFP filter and is visible in the coelomocytes in all larvae (arrowheads). Anterior is to the left, ventral is down. (B-C) Expression of *ets-10p::gfp* between 29 and 40 hours after bleaching at 24°C. For reference, dauer formation occurs at approximately 48 hours after bleaching at 24˚C. All represented timepoints were assessed across two independent experiments (n=118-298 larvae), except the 32 hour timepoint that was assessed in a single experiment (n=61-79 larvae). (B) Nearly all *daf-7* larvae expressed posterior *ets-10p::gfp* at 29 hours after hatching, gradually shifting to full expression over time. (n=61-207 larvae per timepoint). (C) *daf-16; daf-7* larvae expressed no or dim *ets-10p::gfp* at 29 hours. Expression shifted to a mix of dim, full, and non-dauer over time, as some larvae bypassed dauer formation. (n=79-298 larvae per timepoint). **(D-E)** Larvae were sorted to separate plates based on *ets-10p::gfp* expression at 33 hours after hatching at 24˚C. These larvae were examined again at 48 hours after hatching to determine how *ets-10p::gfp* expression had changed. Numbers indicate the number of larvae with the given expression pattern at the indicated time over two independent trials. In this experiment, most *daf-7* larvae **(D)** expressed posterior *ets-10p::gfp* at 33 h and full expression at 48 h. In contrast, most *daf-16; daf-7* larvae **(E)** expressed either no or dim *ets-10p::gfp* at 33h. The larvae with no expression at 33 h were enriched for bypassing dauer formation (non-dauer expression at 48 h), but some dimly expressing larvae also bypassed dauer formation. The few larvae with posterior expression at 33h all showed full expression at 48 h.

## Description

Across species, low insulin/IGF signaling (IIS) activates the Forkhead Box O (FOXO) family of transcription factors to promote diapause formation (Karp 2021) . In favorable environmental conditions, *C. elegans* develops continuously through four larval stages (L1-L4) before reaching reproductive adulthood. In contrast, unfavorable environments such as starvation or overcrowding are sensed in L1 and drive entry into the pre-diapause L2d stage. If conditions improve, L2d larvae molt into L3 and continue development. However, if conditions remain unfavorable, L2d larvae molt into the stress-resistant dauer diapause stage.

The dauer formation decision is mediated by IIS, TGFβ, and nuclear hormone receptor signaling pathways (Baugh and Hu 2020) . Favorable environmental conditions sensed in the L1 lead to expression of several insulins and DAF-7 /TGFβ. High IIS leads to inactivation of the single FOXO protein in *C. elegans* , DAF-16 , and high TGFβ signaling leads to inactivation of the downstream SMAD transcription factor DAF-3 . Low DAF-16 and DAF-3 activity leads to production of dafachronic acid, the ligand for the DAF-12 nuclear hormone receptor. Liganded DAF-12 drives development through the continuous developmental trajectory. In contrast, adverse conditions result in reduced IIS and TGFβ signaling, activation of DAF-16 and DAF-3 , and low dafachronic acid production. DAF-16 , DAF-3 , and ligand-free DAF-12 promote dauer formation (Fielenbach and Antebi 2008; Baugh and Hu 2020) .

Since activation of DAF-16 promotes dauer formation, *daf-16 (0)* null mutants are dauer-defective (Daf-d) (Ogg et al. 1997) . We have previously used a *daf-7 /* TGFβ mutation, *daf-7 ( e1372 ts)* , to drive *daf-16 (0)* larvae into dauer at 24°C (Vowels and Thomas 1992; Karp and Greenwald 2013; Wirick et al. 2021) . However, while some of the *daf-16 (0); daf-7 (ts)* larvae enter dauer, others will bypass dauer formation (Vowels and Thomas 1992; Nika et al. 2016) . This observation raises the question of how L2d is affected in *daf-16 ; daf-7* larvae. Do all larvae that enter dauer develop through an L2d stage equivalent to wild-type larvae in dauer-inducing conditions? Do all larvae that bypass dauer formation also bypass L2d? In addition, the mixed populations of dauer and non-dauer larvae present technical limitations when assessing the role of *daf-16* during dauer. These limitations prompted us to look for tools to predict dauer formation in *daf-16 ; daf-7* larvae in the second larval stage. To address both the developmental and the technical question, we took advantage of a transcriptional reporter of *ets-10* which has been previously described as a tool to visualize L2d and dauer larvae (Shih et al. 2019) . This reporter is particularly useful because it shows the earliest expression among characterized L2d markers, beginning prior to commitment to dauer. Specifically, the expression of *ets-10p::gfp* begins in the posterior intestine of L2d larvae before commitment to dauer entry. The expression increases and spreads anteriorly in the intestine as the larvae commit to dauer and remains high within the dauer stage. By contrast, expression is very low in all non-dauer stages from L1 until early L4. Mid- or late-L4 and adult hermaphrodites show *ets-10p::gfp* expression in the uterus and spermatheca, but at most weak expression in the posterior intestine (Shih et al. 2019) . Furthermore, our existing mRNA-seq data established that *ets-10* mRNA levels were similar in *daf-16 ; daf-7* and *daf-7* dauer larvae (Wirick et al. 2021) .

We first asked if the expression of *ets-10p::gfp* differs between *daf-7* and *daf-16 ; daf-7* mutants during the second larval stage. At 24˚C, dauer formation occurs at approximately 48 hours after egg laying in 100% of *daf-7* animals and in a variable percentage of *daf-16 ; daf-7* larvae (Nika et al. 2016) . To assess the expression of *ets-10p::gfp,* embryos were isolated by bleaching and semi-synchronous populations were incubated at 24°C for varying lengths of time before scoring GFP expression on a fluorescence dissecting microscope. Using the previous report of *ets-10p::gfp* expression in wild-type larvae as a guide (Shih et al. 2019) , we binned expression patterns into five categories ( Fig. 1A ). As previously reported for wild-type, we found that *ets-10p::gfp* expression increased over time in both strains ( Fig. 1B -C). However, *daf-16 ; daf-7* mutants rarely expressed high levels of *ets-10p::gfp* during these times ( Fig. 1C ). At the earliest timepoint assessed (29 h), nearly half of *daf-16 ; daf-7* larvae lacked detectable *ets-10p::gfp* expression whereas nearly all the *daf-7* larvae exhibited posterior expression of the reporter at 29 h. As the populations developed, *daf-7* larvae began to express *ets-10p::gfp* throughout the full intestine. By contrast, *ets-10p::gfp* was expressed dimly in the posterior intestine in most *daf-16 ; daf-7* larvae, with only few animals showing strong intestinal expression.

As expected, part of the *daf-16 ; daf-7* population bypassed dauer formation, which was evident by *ets-10p::gfp* expression in the non-dauer pattern in 100% of larvae that exhibited L4 morphology, namely, larger size and vulval invagination ( Fig. 1C ). Interestingly, we found that the percentage of larvae not expressing the reporter at early timepoints was strikingly similar to the percentage of animals expressing the non-dauer pattern at later timepoints. This observation suggested that the expression levels of *ets-10p::gfp* may be indicative of dauer formation.

To determine if the expression of *ets-10p::gfp* can be used to predict dauer formation, we separated larvae with different expression patterns at early timepoints to observe how *ets-10p::gfp* expression changed over time. We first tightly synchronized populations by collecting hatched L1 larvae for two hours after bleaching. These larvae were incubated at 24°C for a total of 33 hours and then picked to separate plates based on their *ets-10p::gfp* expression. The larvae were returned to 24°C where they continued to develop until 48 hours after bleaching, when they were again assessed for the expression pattern of *ets-10p::gfp* ( Fig. 1D -E). At 33 h, most *daf-7* larvae showed posterior expression, with a few larvae still showing dim expression. By 48 hours, 97% of *daf-7* larvae expressed *ets-10p::gfp* in the full intestine with the remainder showing posterior expression ( Fig. 1D ). Specifically, all larvae with posterior expression at 33 h showed full expression at 48 h. Of the dimly expressing larvae, half showed posterior expression, and the other half showed full expression ( Fig. 1D ). In the *daf-16 ; daf-7* strain, only 46% of larvae exhibited any *ets-10p::gfp* expression at 33h, and most (94%) of that expression was dim ( Fig. 1E ). The expression of *ets-10p::gfp* partially predicted whether the animal would enter dauer. Larvae that did not express *ets-10p::gfp* at 33 h were enriched for larvae that bypassed dauer and expressed *ets-10p::gfp* in the non-dauer pattern at 48 h. Specifically, approximately half (52%) of the non-expressing larvae at 33 h bypassed dauer ( Fig. 1E ). In contrast, larvae that showed any expression of *ets-10p::gfp* early in development were more likely to enter dauer and show full expression of *ets-10p::gfp* at 48 h. Only 14% of larvae with dim expression at 33 h bypassed dauer formation, whereas 0/3 larvae with posterior expression bypassed dauer. Some of the *daf-16 ; daf-7* larvae with full *ets-10p::gfp* expression were visualized with DIC optics to confirm that they exhibited dauer alae. This observation suggests that full *ets-10p::gfp* expression correlates with dauer formation in these mutants. However, we did not use SDS selection or fluorescent beads to verify dauer formation in all such larvae in the population.

In summary, we describe a novel pre-dauer state for *daf-16 ; daf-7* larvae which is characterized by dim *ets-10p::gfp* expression. This dim expression appears to be intermediate between the lack of *ets-10p::gfp* expression observed during the continuous L2 stage and the robust intestinal expression observed during L2d in wild-type or *daf-7* pre-dauer larvae. Furthermore, *daf-16 ; daf-7* larvae that fail to express *ets-10p::gfp* at 33 h after hatching at 24˚C are enriched for bypassing the dauer stage, whereas larvae that express *ets-10p::gfp* at the same timepoint are enriched for entering dauer. It will be interesting to examine if other dauer formation mutants that form partial dauer larvae at incomplete penetrance show similar patterns of *ets-10p::gfp* expression.

## Methods

**Strains and maintenance.**

*C. elegans* strains were maintained at 20°C on NGM (nematode growth media) plates with *E. coli* strain OP50 as a food source (Brenner 1974).

**L2d and Dauer Induction**

Gravid adult hermaphrodites were treated with two 2-minute incubations with a 0.5% v/v Clorox bleach, 1M NaOH solution followed by two dH _2_ O washes to isolate embryos. Embryos were distributed onto NGM plates seeded with OP50 , then incubated at 24°C for the indicated amount of time before assessing the expression of *ets-10p::gfp* in panels B and C.

In panels A, D, and E, to further synchronize the populations after bleaching, embryos were distributed onto unseeded NGM plates and incubated at 24°C for two hours, at which time hatched L1 larvae were transferred to new NGM plates seeded with OP50 . The larvae were then incubated at 24°C until the indicated timepoints, where transfer to seeded plates represented time 2 h.

**Fluorescence Microscopy**

At the indicated timepoints in Figure 1, the expression of *ets-10p::gfp* was visualized and recorded on a Zeiss SteREO Discovery.V12 fitted with M2 Bio for fluorescence equipped with an X-Cite 120Q light source and a GFP/eGFP filter (Kramer Scientific, KSC 296-833D). Images in panel A were captured using an AxioCam mRm Rev 3 camera and Zen 3.2 software. Data shown in panels B-E are from larvae on NGM plates, but images in panel A were taken of larvae mounted on a 2% agarose pad on a glass slide, anesthetized in 0.1M levamisole and imaged using a 10X objective at 40X zoom. In panels D-E, synchronized larvae were separated into categories based on their expression pattern of *ets-10p::gfp* 33 hours after synchronization at the embryo stage, incubated at 24°C until 48 hours after synchronization and their expression pattern of *ets-10p::gfp* was recorded.

## Reagents

**Table:** 

Strain name	Genotype
XV273	*daf-7 ( e1372 ) III; syIs601 [ets-10p::gfp + ofm-1p::rfp] *
XV279	*daf-16 ( mgDf50 ) I; daf-7 ( e1372 ) III; syIs601 [ets-10p::gfp + ofm-1p::rfp] *
